# In Vitro Antagonistic Activity of Plant Growth Promoting Rhizobacteria Against Aggressive Biotypes of the Green Mold

**DOI:** 10.1002/jobm.202400422

**Published:** 2024-10-03

**Authors:** Baran Mis, Kemal Karaca, Rengin Eltem

**Affiliations:** ^1^ Department of Bioengineering, Graduate School of Natural and Applied Sciences Ege University Izmir Turkey; ^2^ Department of Bioengineering, Faculty of Engineering Ege University Izmir Turkey

**Keywords:** *Agaricus bisporus*, *Bacillus* spp, biological control, *Pseudomonas* spp, *Trichoderma aggressivum*

## Abstract

During the cultivation of button mushrooms, the green mold epidemic, which causes a decrease in productivity, is a very important problem. The environmental harm of chemicals used in the control of such epidemics and the demand of consumers for organic products without chemicals have brought environmentally friendly biological control to the fore. Biological control can be achieved by the use of antagonistic microorganisms and their metabolites. In this study, the effectiveness of *Bacillus* spp. and *Pseudomonas* spp. for the biological control of the aggressive biotypes of the green mold disease agent *Trichoderma aggressivum* strains was examined in vitro. For this purpose, the antifungal effects of *Bacillus* spp. and *Pseudomonas* spp. against *T. aggressivum* strains were examined by in vitro dual culture test. Afterward, the antifungal activity of *Bacillus* spp. metabolites was assessed further using the agar well diffusion method. Then, it was determined whether the bacterial strains showing antifungal activity showed antagonistic activity against *A. bisporus*. Although none of the *Pseudomonas* spp. showed antifungal activity against *T. aggressivum* strains, most of the *Bacillus* spp. were found to have high activity. It has been concluded that *Bacillus* sp. Ö‐4‐82 may be potential biological control agent for button mushroom cultivation.

**Abbreviations**
CLP: cyclic lipopeptide.
LB: Luria–Bertani
NB: nutrient broth
PDA: potato dextrose agar
PGPR: plant growth promoting rhizobacteria
SDB: Sabouraud dextrose broth

## 1 Introduction

Although 90% of its total weight is water, *Agaricus bisporus*, commonly known as a button mushroom, contains high amounts of vitamins and proteins, making it a valuable asset with a large share of the mushroom market used as both food and medicine for humans [1, 2]. As with all agricultural products, *A. bisporus* is affected by many biotic and abiotic factors, and therefore, decreases in crop yield may occur. One of the major threats to button mushroom cultivation, which can be affected by biotic factors such as fungi, bacteria, viruses and nematodes, is green mold disease caused by a wide range of *Trichoderma* species (e.g., *T. aggressivum, T. harzianum, T. atroviride, T. koningii, T. virens, T. mienum*) [3, 4, 5]. Green mold disease, which was first identified in 1953, has been reported in various countries over time and has been proven to cause crop losses of up to 100% [6, 7]. Aggressive green mold biotypes are identified in Europe and North America, respectively, as *T. aggressivum f. europaeum and T. aggressivum f. aggressivum* [8, 9, 10]. *T. aggressivum* strains are considered a disease agent since *Trichoderma* species compete with other organisms for nutrients in the environment and show mycoparasitic properties by breaking down the cell walls of other organisms in the environment with the lytic enzymes (β‐glucanase, chitinase, cellulase, protease, and so on) they secrete [11, 12].

Chemical fungicides that inhibit the growth of *Trichoderma* are used to prevent green mold disease during button mushroom cultivation. However, since *A. bisporus* mycelia are sensitive to chemicals and are affected quickly, the use of chemical pesticides reduces the efficiency of button mushroom production. With the realization of the harmful effects of chemical pesticides on nature and consumers, people' preferences have changed to move away from these pesticides. In addition to harmful side effects, cost also limits pesticide use. In line with the new legal regulations brought by our changing world and the demands of consumers, chemical use must be changed to an alternative approach. Since biological control is a more sustainable method than chemical control, it is thought that the best alternative method is the use of biological control microorganisms and/or the metabolites of these microorganisms. *Bacillus* and *Pseudomonas* are the most widely used biological control agents for bacterial strains because of their properties, such as lytic enzyme production, induction of plant resistance and antibiosis [3, 13, 14, 15, 16, 17].

Within the scope of this study, the antifungal effects of *Bacillus* spp. and *Pseudomonas* spp., which can be used as biocontrol agents against green mold disease caused by *Trichoderma aggressivum f. aggressivum* and *Trichoderma aggressivum f. europaeum* in the cultivation of the button mushroom *Agaricus bisporus*, were examined.

## 2 Materials and Methods

### 2.1 Microorganisms

*Bacillus* (*n* = 92) sp. and *Pseudomonas* (*n* = 52) sp., which have antifungal activity and plant growth‐promoting rhizobacteria (PGPR) properties, and *T. aggressivum f. aggressivum* and *T. aggressivum f. europaeum* isolated from casing soil and mushroom compost, which have the potential to cause green mold disease in button mushroom cultivation, found in the Industrial Microbiology Laboratory Collection of the Bioengineering Department of Ege University were used. *Agaricus bisporus* spawn were obtained from a commercial enterprise [7, 18, 19, 20, 21, 22, 23].

### 2.2 Evaluation of in Vitro Antagonistic Activity

#### 2.2.1 Determination of Antifungal Effect by Dual Culture

A loopful of streaked *Bacillus* spp. and *Pseudomonas* spp. and agar discs (6 mm) taken from the active *Trichoderma* culture placed 5 cm apart were incubated in Petri dishes containing potato dextrose agar (PDA) at 30°C for 4 days to determine antagonistic activity. The test was performed in triplicate. Agar plates with only *Trichoderma* discs were used as controls [3, 24].

The following formula was used to calculate the % growth inhibition:

$$\%\;\mathrm{Growth}\;\mathrm{Inhibition}=\frac{C-T}{C}x100.$$

C: Growth diameter of *Trichoderma* colonies in control Petri dishes, T: Growth diameter of the *Trichoderma* colony in the Petri dish tested for antifungal activity.

#### 2.2.2 Determination of Antifungal Effect by Agar Well Diffusion Method

Two percent inoculum taken from bacterial cultures prepared by incubation in sterile nutrient broth (NB) medium in a shaking incubator at 30°C for 18 h at 180 rpm was transferred to 50 mL of Luria–Bertani (LB) broth and incubated in a shaking incubator at 180 rpm and 30°C for 72 h [25, 26, 27, 28]. After production, the bacterial cultures were centrifuged at 9000 rpm for 10 min, and the pH of the supernatant obtained was reduced to 2.0 with 6 N HCl and kept at +4°C for 1 night. After centrifugation at 10,000 rpm for 10 min, the supernatant was removed, and 2 mL of methanol was added to the resulting pellet and kept at +4°C for 1 additional night. This process was repeated once more to obtain cyclic lipopeptide (CLP) extract. The extracts in methanol were passed through a sterile syringe filter with a 0.45‐µm pore diameter and stored at −20°C [29, 30, 31, 32]. Wells with a diameter of 6 mm were opened with the help of a hollow sterile glass rod in petri dishes containing PDA to determine the antagonistic effect. One hundred microliters of extracts were added to these wells, and agar disks taken from the active *Trichoderma aggressivum* colony were placed 5 cm opposite them. For the control, plates containing only *T. aggressivum* were used. After 4 days of incubation, antifungal activity was calculated according to the % growth inhibition formula [33].

### 2.3 Mycelial Growth Inhibition Tests

Based on the agar well diffusion method, fermentation liquids of 8 *Bacillus* spp. produced in LB broth medium were used to confirm their antifungal effects against the *Trichoderma aggressivum* with mycelial growth inhibition test (Supporting Information S1: Tables S1 and S2). Fermentation liquids of *Bacillus* spp. produced in LB broth medium were separated from the pellet by centrifugation at 10,000 rpm for 10 min, sterilized with the help of a sterile 0.45‐µm syringe filter and stored at −20°C [34].

To examine the antifungal effect of culture filtrates against *Trichoderma aggressivum* strains, a spore suspension (1 × 10^6^ spores/mL) was prepared by gently scraping the spores with a loop using 0.01% Tween 80 solution from *Trichoderma* strains grown in horizontally prepared PDA, inoculated at 2% into flasks containing 50‐mL Sabouraud dextrose broth (SDB) and incubated for 24 h at 30°C with stirring at 180 rpm. Then, 5 mL of previously prepared bacterial culture filtrates was added to this medium, and the mixture was incubated for another 72 h under the same conditions. Sterile liquid medium was added to the control flasks instead of the culture filtrate. At the end of the incubation, the *T. aggressivum* biomass was separated from the liquid parts by filtration with the help of a vacuum pump, and the wet weight of the samples was measured. The drying process required for dry weight determination was carried out between 60°C and 70°C. Weight measurements were performed until the dry weight of the samples did not change [35].

To examine the antifungal effect of culture filtrates on *A. bisporus*, five agar discs were removed from the fungus colony activated on the PDA plate with a hollow sterile glass rod and transferred to an Erlenmeyer flask containing 50 mL of complete yeast media (CYM) broth. Bacterial culture filtrates were added to the *A. bisporus* production media, which were incubated in static culture at 25°C for 7 days and incubated for another 14 days under the same conditions. Liquid medium was added to the control flasks instead of the culture filtrate. At the end of a total of 21 days of incubation, the *A. bisporus* biomass was separated from the liquid part with the help of a vacuum pump. Its wet weight was measured, and its dry weight was subsequently measured by drying at 60–70°C [35].

### 2.4 Statistical Analysis

All experiments were performed in triplicates. Means and standard deviations were calculated statistically. Statistical analyses were performed using the PASW (SPSS) Statistic v.18 package program and analysis of variance (ANOVA) according to the Tukey test (*p* < 0.05).

## 3 Results

### 3.1 Evaluation of in Vitro Antagonistic Activity

Antifungal activities of 92 *Bacillus* spp. and 52 *Pseudomonas* spp. against *Trichoderma aggressivum* strains were first determined by dual culture tests and thereafter only 21 *Bacillus* spp. producing CLPs with inhibition rates of 70% or over against *T. aggressivum* were also examined through the agar well diffusion method.

#### 3.1.1 Determination of Antifungal Effect by Dual Culture

**Figure 1:**
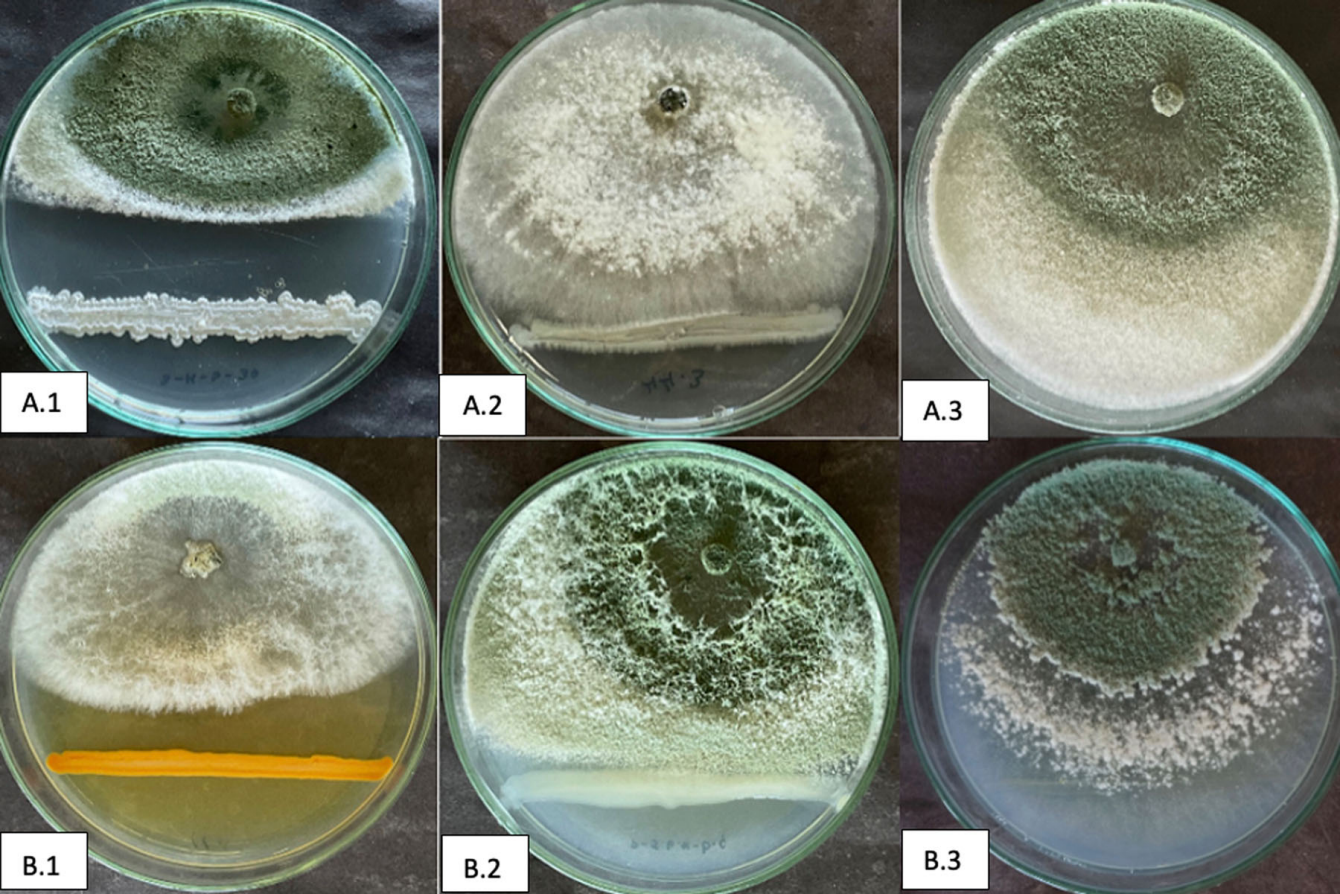
Different growth patterns observed in the dual culture test. *Trichoderma aggressivum f. europaeum* against; (A.1) *Bacillus* sp. 3‐K‐S‐37 (inhibition zone formation), (A.3) *Bacillus* sp. Ö‐4‐13‐a (overgrown growth), (B.1) *Pseudomonas* sp. 168 (inhibition zone formation), (B.2) *Pseudomonas* sp. P‐5.6. *‐b‐c (deadlocked growth), and *T. aggressivum f. aggressivum* against; (A.2) *Bacillus* sp. 44.3 (deadlocked growth), (B.3) *Pseudomonas* sp. P‐7.7. *‐1 (overgrown growth).

Three different growth types (Figure 1) were observed as a result of the effect of both *Bacillus* spp. and *Pseudomonas* spp. on the growth of *T. aggressivum* strains. The dual culture test was performed. In control Petri dishes, *T. aggressivum* strains grew to cover the entire petri dish. In experiments where growth similar to that of the control plates was observed, *Pseudomonas* spp. and *Bacillus* spp. were not able to stop the pathogen, so *T. aggressivum* strains overgrown on bacterial cultures. However, some *Bacillus* spp. and *Pseudomonas* spp. achieved deadlocked growth by stopping *T. aggressivum* strains at the point it encountered the pathogen. In cases where the growth of *T. aggressivum* strains was stopped before encountering the bacteria, and inhibition zones were formed. However, this type of growth is generally caused by *Bacillus* spp. and few *Pseudomonas* spp. (Figure 1).

When the results of the dual culture test were examined, a wide range of inhibition percentages were determined for *Bacillus* and *Pseudomonas* species that have antifungal effects. It has been determined that *Bacillus* spp. have greater antifungal activity against *T. aggressivum* strains, the agent of green mold disease in cultivated mushroom cultivation, than *Pseudomonas* spp. Although a large majority of the 92 *Bacillus* spp., which were determined to produce lytic enzymes such as protease, cellulase and chitinase, and antifungal metabolites such as siderephore and HCN [20], showed strong inhibitory effects, some of them could not prevent or even slow the growth of *Trichoderma*. An inhibition rate of 15% ± 6.43%–83.18% ± 0.93% was achieved against *T. aggressivum f. aggressivum*, and *Bacillus* sp. Ö‐1‐57‐b had the highest antagonistic activity, with an inhibition rate of 83.18% ± 0.93%. An inhibition rate of 0%–79.29% ± 6.43% was achieved against *T. aggressivum f. europaeum*, and the highest antagonistic activity was obtained in *Bacillus* sp. T‐4‐17, with an inhibition rate of 79.29% ± 6.43%. Only 22 of 92 *Bacillus* spp. showed an inhibition percentage of 70% or more. The numerical distribution of the antagonistic bacteria used in the dual culture test according to their percentage inhibition rates against both *Trichderma* aggressivum strains are shown in Tables 1 and 2.

**Table 1:** Numerical distribution of *Pseudomonas* spp. affecting *Trichoderma* strains according to percentage inhibition rates.

Inhibition percent range (%)	*T. aggressivum f. agressivum*	*T. aggressivum f. europaeum*
	Number *Pseudomonas* spp. with antagonistic effect (*n*)	
0–19	37	41
20–29	5	5
30–39	5	1
40–50	5	5

**Table 2:** Numerical distribution of *Bacillus* spp. affecting *Trichoderma* strains according to percentage inhibition rates.

Inhibition percent range (%)	*T. aggressivum f. agressivum*	*T. aggressivum f. europaeum*
	Number *Bacillus* spp. with antagonistic effect (*n*)	
0–49	9	13
50–59	2	47
60–69	62	29
70–90	19	3

Among the 52 *Pseudomonas* sp. strains examined, an inhibition rate of 0%–46.50% ± 0.72% was achieved against *T. aggressivum f. aggressivum*, and *Pseudomonas* sp. 39 showed the highest antagonistic activity, with 46.50% ± 0.72%. With respect to *T. aggressivum f. europaeum*, an inhibition rate of 0%–46.43% ± 3.57% was achieved, and the highest antagonistic activity was achieved by *Pseudomonas* sp. 176, with 46.43% ± 3.57%.

#### 3.1.2 Determination of Antifungal Effect by Agar Well Diffusion Method

The antifungal activity of 21 *Bacillus subtilis* strains [20], which are known to produce CLPs [36] and exhibit an inhibition rate of 70% or more against *T. aggressivum* strains according to dual culture tests, was examined by the agar well diffusion method, and the results are given in Table 3.

**Table 3:** Examination of antifungal metabolites produced by *Bacillus* spp. by the agar well diffusion method against *T. aggressivum* strains.

Inhibition rate (%)		
Codes	*T. aggressivum f. agressivum*	*T. aggressivum f. europaeum*
*Bacillus* sp. T‐1‐18	16 ± 1^i^	21.67 ± 1.18^cde^
*B. subtilis* T‐2‐2	22 ± 1^efgh^	31.67 ± 1.67^b^
*Bacillus* sp. T‐3‐10	21 ± 1^fghi^	35 ± 1.67^b^
*Bacillus* sp. T‐3‐12‐b	25.23 ± 0.9^cdef^	20.39 ± 0.97^def^
*Bacillus* sp. T‐4‐13‐a	21 ± 1^fghi^	32.5 ± 0.83^b^
*Bacillus* sp. T‐4‐17	20 ± 1^ghi^	42.5 ± 0.83^a^
*Bacillus* sp. Ç‐2‐30‐a	24.32 ± 0.9^defg^	15.53 ± 0.97^fg^
*B. subtilis* Ç‐2‐30‐b	17.12 ± 1.8^hi^	2.91 ± 0.97^h^
*Bacillus* sp. Ö‐1‐57‐b	36.94 ± 0.9^a^	34.95 ± 2.91^b^
*B. subtilis* Ö‐1‐59‐b	29.73 ± 1.8^bc^	24.27 ± 0.97^cd^
*B. subtilis* Ö‐4‐57‐b	30.63 ± 0.9^b^	26.21 ± 1.94^c^
*Bacillus* sp. Ö‐4‐82	30.63 ± 0.9^b^	24.27 ± 0.97^cd^
*Bacillus* sp. Ö‐5‐1	27.03 ± 0.9^bcde^	20.39 ± 3.88^def^
*Bacillus* sp. B‐2‐6‐b	17.12 ± 1.8^hi^	10.68 ± 1.94^g^
*Bacillus* sp. K‐6‐22	24.32 ± 3.6^defg^	18.45 ± 1.94^ef^
*Bacillus* sp. K‐7‐14‐1	27.93 ± 0.9^bcd^	22.33 ± 0.97^cde^
*B. subtilis* 1‐K‐49‐a	26.13 ± 1.8^bcde^	22.33 ± 0.97^cde^
*Bacillus* sp. 2‐K‐13‐a	25.23 ± 2.7^cdef^	17.48 ± 0.97^ef^
*Bacillus* sp. 3‐K‐S‐17‐a	23.42 ± 2.7^defg^	19.42 ± 0.97^def^
*Bacillus* sp. 3‐K‐S‐39‐a	22.52 ± 1.8^defg^	21.36 ± 2.91^cde^
*B. subtilis* 4‐Ka‐59‐a‐a	26.12 ± 0.9^bcde^	24.27 ± 0.97^cd^

*Note:* Values followed by different letters are significantly different according to the Tukey test (*p *= 0.05)

**Figure 2:**
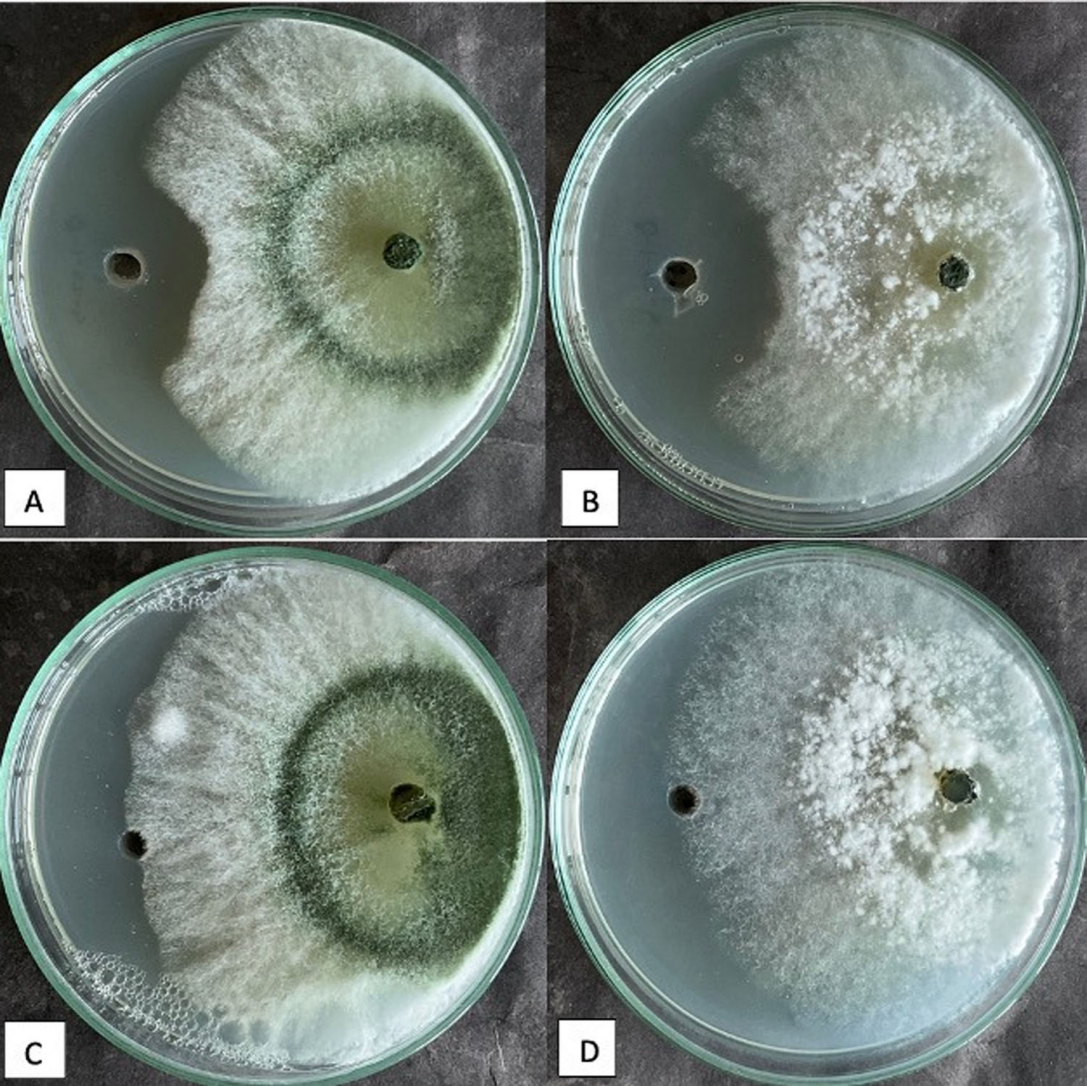
Inhibition zones formed by *Bacillus* sp. Ö‐1‐57‐b against the (A) *T. aggressivum f. europaeum* strain and (B) *T. aggressivum f. aggressivum* strain. Control Petri plates of (C) *T. aggressivum f. europaeum* strain and (D) *T. aggressivum f. aggressivum* strain.

*Bacillus* sp. T‐4‐17 showed the highest antagonistic activity against the *T. agaggresivum f. europaeum* strain, with an inhibition rate of 42.5%. Figure 2 shows that *Bacillus* sp. Ö‐1‐57‐b had the highest percentage inhibition rates against *T. aggressivum f. europaeum* (36.94%) and *T. aggressivum f. aggressivum* (34.95%). These two *Bacillus* strains also showed the greatest reduction in inhibition rates in the dual culture test. However, the percentage inhibition rates obtained in the agar well method are not as high as the rates obtained in the dual test because there are no lytic enzymes with antifungal activity, such as protease, chitinase, cellulase, and β‐1,3‐glucanase, or antifungal metabolites, such as siderephore and HCN, in the medium, only CLPs.

Of the 21 *Bacillus* sp. strains examined, *T. aggressivum f. aggressivum* 18 had antagonistic effects in the range of 15%–29%, and three had an inhibitory effect of 30%–45%; *T. aggressivum f. europaeum* had antagonistic effects in the range of 2.91%–14%, 14 had antagonistic effects in the range of 15%–29%, and five had antagonistic effects in the range of 30–45%. The highest antifungal activity was observed for *Bacillus* sp. T‐4‐17 (42.5% ± 0.83%) against *T. aggressivum f. europaeum* and for *Bacillus* sp. Ö‐1‐57‐b (36.94% ± 0.9%) against *T. aggressivum f. aggressivum*. The antagonistic activity identified is thought to be CLPs produced by *Bacillus* spp. Most of the *Bacillus subtilis, B. amyloliquefaciens, B. mojavensis, B. velezensis, and B. methylotrophicus* strains show antagonistic effects by producing secondary bioactive CLPs [37, 38].

### 3.2 Mycelial Growth Inhibition Tests

The experiments were continued with eight *Bacillus* spp., which were significantly different (*p* < 0.05) from the values obtained in the statistical analysis performed via the agar well diffusion method (Table 3), which was carried out to determine the antifungal effect against *Trichoderma aggressivum* strains of *Bacillus* spp. (*n* = 21) and had an inhibition rate of 30% or more.

Biomass reduction rates as a result of examining the effect of culture filtrates obtained from the *Bacillus* spp. production media on the mycelial growth of *T. aggressivum* strains and *Agaricus bisporus* are given in Table 4. The biomass reduction rate of *T. aggressivum f. aggressivum* was 20.37%–72.22%, while this rate was between 2.56% and 53.84% in *T. aggressivum f. europaeum*. A biomass reduction rate of 22.86%–91.43% was detected for *A. bisporus*. Although *Bacillus* spp. reduced the growth of *Trichoderma aggressivum f. aggressivum* and *Trichoderma aggressivum f. europaeum*, which are agents of green mold disease in button mushroom cultivation, these strains also reduced the growth of *A. bisporus* (Table 4).

**Table 4:** Effect of *Bacillus* spp. culture filtrates on the growth of *T. aggressivum* strains and *A. bisporus*.

	*T. aggressivum f. aggressivum*		*T. aggressivum f. europaeum*		*Agaricus bisporus*	
Codes	Dry weight (g/50 mL)	Biomass reduction rate (%)	Dry weight (g/50 mL)	Biomass reduction rate (%)	Dry weight (g/50 mL).	Biomass reduction rate (%)
*B. subtilis* T‐2‐2	0.15 ± 0.25^b^	72.22	0.38 ± 0.03^a^	2.56	0.03 ± 0.02^c^	91.43
*B. subtilis* Ö‐4‐57‐b	0.27 ± 0.01^ab^	50.0	0.18 ± 0.01^b^	53.84	0.12 ± 0.02^bc^	65.71
*Bacillus* sp. T‐3‐10	0.29 ± 0.25^ab^	46.3	0.34 ± 0.06^a^	12.82	0.05 ± 0.07^c^	85.71
*Bacillus* sp. T‐4‐13‐a	0.43 ± 0.03^ab^	20.37	0.33 ± 0.02^ab^	15.38	0.05 ± 0.04^c^	85.71
*Bacillus* sp. T‐4‐17	0.31 ± 0.18^ab^	42.59	0.37 ± 0.08^a^	5.13	0.12 ± 0.01^bc^	65.71
*Bacillus* sp. Ö‐1‐57‐b	0.28 ± 0.03^ab^	48.15	0.29 ± 0.11^ab^	25.64	0.08 ± 0.04^bc^	77.14
*Bacillus* sp. Ö‐4‐82	0.22 ± 0.22^ab^	59.26	0.31 ± 0.03^ab^	20.51	0.27 ± 0.17^ab^	22.86
Control	0.54 ± 0.01^a^	—	0.39 ± 0.01^a^	—	0.35 ± 0.04^a^	—

*Note:* Values followed by different letters are significantly different according to the Tukey test (*p* = 0.05). The biomass reduction rate is not valid for control values.

The examined *Bacillus* spp. culture filtrates caused average biomass reductions of 48.41% for *T. aggressivum f. aggressivum*, 19.41% for *T. aggressivum f. europaeum* and 70.61% for *A. bisporus*. *Bacillus* sp. Ö‐4‐82 showed a biomass reduction of 59.26% against *T. aggressivum f. aggressivum* and 20.51% against *T. aggressivum f. europaeum*, one of the most common green mold disease agents in cultivated mushroom cultivation [7], and 22.86% against *A. bisporus*. This bacterium has the potential to be used as a biocontrol agent against green mold disease in button mushroom cultivation.

## 4 Discussion

When the current literature is examined, it becomes clear that the number of PGPR strains tested and giving various results and the number of in vitro antagonistic tests used are quite limited compared to our own study. In a study examining green mold disease in button mushrooms, the antagonistic effects of *Bacillus subtilis* MSG‐5, *B. amyloliquefaciens* SGM‐1, *B. amyloliquefaciens* SGM‐2, *Stenotrophomonas maltophilia* MSG‐11, and *Pseudomonas rhodesiae* MSG‐15 strains against *T. aggressivum f. aggressivum* were examined using dual culture tests, and after 7 days of incubation, *P. rhodesia* MSG‐15 (46.6%) showed the greatest inhibitory effect on *T. aggressivum f. aggressivum*, while the lowest inhibitory effect (38.61%) was obtained for *B. amyloliquefaciens* SGM‐2 [3]. Of the 92 *Bacillus* spp. examined in our study, 88 inhibited *T. aggressivum f. aggressivum* by more than 38%, and 84 inhibited *T. aggressivum f. europaeum by more than 38%*. All 52 *Pseudomonas* spp. examined showed a percentage inhibition rate below 46%. In another study, a dual culture test was applied to determine the antagonistic effects of *fluorescent Pseudomonas* and *Bacillus* sp. against *Trichoderma* sp. It was determined that both bacteria examined had antagonistic effects against *Trichoderma*, but no significant difference was detected in the inhibition zones formed between *Bacillus* sp. (13.6 mm) and *fluorescent Pseudomonas* (15.5 mm) [39]. *Pseudomonas* and *Trichoderma* species are known to grow together [40]. In a study examining the in vitro antifungal effect of *Pseudomonas tolaasii* and *Pseudomonas putida* isolated from mushroom compost [35], it was observed that *P. putida* culture supernatant increased *T. aggressivum* mycelial growth by 44%.

It has been proven that *Trichoderma* species are natural decomposition agents and biological agents for bioremediation [41]. Various *Trichoderma* strains are among the most resistant microorganisms to natural and synthetic chemicals and toxins and are able to rapidly degrade compounds such as hydrocarbons, chlorophenolic compounds, polysaccharides and pesticides [42, 43, 44, 45, 46, 47]. In our study, it was observed that during the 4‐day incubation period, *T. aggressivum f. europaeum* had faster spreading and sporulating growth than did *T. aggressivum f. aggressivum* due to the possibility of its ability to break down the antifungal metabolites created by *Bacillus* spp. In a study in which the highest recommended dosage of four different commercial fungicides (25 g fludioxonil + 10 g metalaxyl‐M, 360 g/l hymexazol, 53.8% copper hydroxide + tetrasodium pyrophosphate and 250 g/l azoxystrobin) were examined against *Trichoderma atroviride* (*n* = 5), *T. citrinoviride* (*n* = 6), and *T. harzianum* (*n* = 2) under in vitro conditions, it was concluded that the examined *Trichoderma* spp. showed different growth and/or growth inhibition due to their ability to degrade fungicides [48].

Any plant growth promoting rhizobacterium with high inhibition rate against the aggressive biotypes of the green mold is meaningless as long as it has not low inhibition against *Agaricus bisporus*. In a study by Kosanovic et al. [35], in which the antifungal effect of *Bacillus velezensis* extract was examined against *A. bisporus* and *Trichoderma aggressivum f. europaeum*, this extract was added at 25% (v/v) to media containing *Trichoderma* and *A. bisporus*, which were incubated alone for 48 h and 8 days, respectively, and incubation was continued for 24 and 48 h, respectively. After incubation, the wet mycelial weights obtained from the flasks containing *T. aggressivum f. eurpaeum* and *A. bisporus* to which *Bacillus* extract was added were 2.9 ± 0.3 g/50 ml and 5.1 ± 0.7 g/50 mL, respectively, while they were 4.9 ± 1.0 g/50 mL and 5.2 ± 1.2 g/50 mL, respectively, in the flasks in which no bacterial extract was added. The bacterial extract used in the present study downregulated proteins that play a role in the survival of *A. bisporus*, signaling proteins, ribosomal proteins and proteins that play a role in secondary metabolism. However, the downregulation of these proteins does not have a negative effect on *A. bisporus*, and *B. velezensis* can be used as a potential biological control agent. Through morphological alterations such as spore germination suppression, mycelial growth inhibition, spore and hyphal tip rupture, and germ tube elongation, phytopathogenic fungi are suppressed by antagonistic bacterial lytic enzymes [49]. Previous studies have shown that *Bacillus* culture supernatants containing the abovementioned lytic enzymes inhibit the mycelial growth of various fungal pathogens [49, 50, 51, 52]. The amount of *Aspergillus niger* biomass that was incubated in liquid media for 60 h ranged from 0.37 g/50 mL–0.49 g/50 mL, but when the bacteria were incubated with fermentation filtrates containing lytic enzymes (chitosanase, chitinase, N‐acetyl‐β‐hexoaminidase, and protease) secreted by six different *Bacillus* species, the biomass reduction decreased to 0.28–0.43 g/50 mL [53]. In our study, at the end of incubation of *T. aggressivum f. aggressivum, T. aggressivum f. europaeum* and *A. bisporus* with *Bacillus* sp. culture filtrates, the wet micelle weights were 8.53 ± 3.84–13.79 ± 7.35 g/50 mL, 11.97 ± 4.07–17.93 ± 3.43 g/50 mL, and 8.12 ± 3, respectively. The dry micelle weights were 0.15 ± 0.25 g/50 mL, 0.18 ± 0.01 g/50 mL, and 0.03 ± 0.02 g/50 mL; in flasks without added culture filtrate, the wet mycelia weights were 21.37 ± 2.69 g/50 mL, 18.40 ± 1.63 g/50 mL, and 17.24 ± 2.06 g/50 mL; and the dry micelle weights were 0.54 ± 0.01 g/50 mL, 0.39 ± 0.01 g/50 mL, and 0.35 ± 0.04 g/50 mL, respectively.

The results of the study indicated that the *Bacillus* sp. Ö‐4‐82 strain selected through laboratory‐scale experiments shows potential for managing aggressive biotype of *T. aggressivum f. aggressivum* not for the other biotype of *T. aggressivum f. europaeum* due to low antifungal effect (20.51% biomass reduction). However, in vivo studies are necessary to validate its antifungal efficacy against green mold. According to these studies, it has become clear that biological control is important for *A. bisporus* cultivation, and studies in this field will contribute to the literature.

## Author Contributions

**Baran Mis:** conceptualization (equal), writing–original draft (equal), writing–review and editing (equal), project administration, funding acquisition, visualization (equal). **Kemal Karaca:** conceptualization (equal), writing–original draft (equal), writing–review and editing (equal), software,visualization (equal). **Rengin Eltem:** supervision, validation, conceptualization (equal), writing–original draft (equal), writing–review and editing (lead).

## Conflicts of Interest

The authors declare no conflicts of interest.

## Supporting information

Supporting information.

## Data Availability

The data that supports the findings of this study are available in the supplementary material of this article.
