# Supplementary material for: In Vitro Antagonistic Activity of Plant Growth Promoting Rhizobacteria Against Aggressive Biotypes of the Green Mold
Source: J Basic Microbiol. 2024 Oct 3;64(12):e2400422. doi: 10.1002/jobm.202400422 (PMC11609499; doi:10.1002/jobm.202400422)
Supplement: Supplementary file 1 — Supporting information. [file JOBM-64-e2400422-s001.doc]

**SUPPORTTING INFORMATION**

**Table S1.** Growth Inhibition Ratios of Bacillus spp. against Trichoderma strains.

**% Growth Inhibition**

***Bacillus* sp. *T. aggressivum f. agressivum T. aggressivum f. europaeum***

**T-1-18**   **69,29±2,13 78,57±2,86**

**T-1-18-a**  63,57±0,72 65±2,14

**T-2-2 (ON722567)** **71,4±1,43 61,43±1,43**

**T-2-16** 65,71±1,43 61,43±1,43

**T-3-10**  **71,43±1,43 52,86±1,43**

**T-3-12-a (ON722570)** 68,7±0,76 57,86±2,14

**T-3-12-b**  **70±0,72 60,71±0,72**

**T-4-8 (ON722557)** 61,68±0,93 62,14±2,14

**T-4-9**  69,29±1,47 63,57±2,14

**T-4-13-a**  **71,43±0,41 57,14±0,72**

**T-4-14-a (ON722561)**  62,62±0,93 57,14±2,86

**T-4-14-b** 68,57±1,43 57,14±4,44

**T-4-15**  69,29±0,71 60±1,83

**T-4-17**  **66,43±0,72 79,29±6,43**

**T-4-19**  68,57±0,42 57,14±0,72

**T-4-20-e-a**  62,6±0,76 60±2,86

**T-11-8** 67,86±0,72 53,57±0,72

**Ç-1-16-a**  69,29±2,14 56,43±0,71

**Ç-2-30-a**  **70±0,72 60±1,43**

**Ç-2-30-b (ON722558) 79,44±1,87 58,57±1,43**

**Ç-2-36** 60,71±0,72 50±1,43

**Ç-3-10 (ON722559)** 63,36±0,76 61,43±1,43

**Ç-3-15-a**  62,86±1,43 55±2,14

**Ç-3-19** 66,43±0,72 50±0,71

**Ç-3-20-a**  **(ON722568)** 67,29±0,93 58,57±2,86

**Ç-3-22-a** 69,29±0,72 55,71±1,43

**Ç-3-22-b**  65±0,71 55±0,71

**Ç-3-23** 66,43±0,72 56,43±0,72

**Ç-3-31** 67,14±1,43 55,71±1,43

**Ö-1-31-b** 68,57±0,71 57,86±0,71

**Ö-1-57-b** **83,18±0,93 65±0,71**

**Ö-1-59-b (ON722560)** **71,03±2,80 57,14±1,43**

**Ö-1-85 (ON722553)** 64,12±0,76 56,43±0,71

**Ö-2-38** 68,22±5,61 65,71±2,86

**Ö-2-44-b** 62,86±0,72 66,43±0,72

**Ö-2-55-a** 64,12±0,76 59,29±0,71

**Ö-2-61-a** 67,86±0,72 56,43±0,72

**Ö-2-61-c**  68,57±1,43 57,86±0,72

**Ö-3-5-b**  65±0,71 55±0,71

**Ö-3-21 (ON722552)** 63,57±0,71 58,57±1,43

**Ö-3-30** 62,6±0,76 54,29±1,43

**Ö-4-11-a** 64,89±0,76 45,71±1,43

**Ö-4-13-a** 50,47±4,67 -

**Ö-4-57-a** 64,89±1,53 57,86±0,71

**Ö-4-57-b** **(ON722566)** **80,37±0,93 64,29±5,71**

**Ö-4-68 (ON722569)** 65,65±2,29 59,29±0,71

**Ö-4-82** **79,44±1,87 57,14±1,43**

**Ö-5-1** **82,24±0,93 61,43±2,86**

**Ö-6-50-a** 69,29±2,14 57,86±0,71

**B-2-6-b**  **71,96±1,87 59,29±2,14**

**B-2-24-a-b**  53,27±0,93 47,14±0,71

**K-6-13-b-a**  67,14±1,43 60,71±3,57

**K-6-22**  **70,09±3,74 70±1,43**

**K-7-14-1 79,44±0,93 60±1,43**

**K-9-37-a (ON722555)** 64,89±0,76 59,29±0,71

**A-1 (ON722554)**  63,36±0,76 57,86±0,71

**1-K-49-a (ON722565)** **81,31±3,74 66,43±2,14**

**2-K-5** 68,57±0,71 55,71±1,43

**2-K-13-a 78,5±4,67 60±1,43**

**2-K-29 (ON722563)** 64,12±0,76 57,86±0,71

**2-K-35-a** 63,36±0,76 54,29±1,43

**2-K-37** 66,43±0,71 61,43±1,43

**3-K-10** 67,86±0,71 62,86±2,86

**3-K-25** 67,14±1,43 60±0,71

**3-K-S-1-a** 61,83±0,76 56,43±2,14

**3-K-S-14** 65,71±0,71 60±2,86

**3-K-S-17-a** **76,64±0,93 58,57±1,65**

**3-K-S-37** 68,57±0,71 62,14±0,71

**3-K-S-39-a** **71,43±0,71 57,14±0,71**

**3-K-S-47-a** 65±0,71 60,71±0,71

**3-K-S-49** 63,57±2,14 60,71±0,71

**3-K-S-60-a** 65±0,71 54,29±0,71

**3-K-S-61** 67,14±0,71 59,29±0,71

**3-K-S-64** **(ON722562)** 68,57±0,71 62,86±0,71

**4-Ka-22 (ON722556)**  67,14±1,43 60,71±0,71

**4-Ka-58-1** 60,75±0,93 54,29±1,43

**4-Ka-59-a-a (ON722564)** **71,4±1,43 66,43±5**

**B3P5** 60±0,71 56,43±0,71

**16+** 62,14±0,71 58,57±1,43

**10+** 69,29±0,71 57,86±0,71

**42.3** 62,14±2,14 59,29±2,14

**44.3** 35±0,71 15,71±2,86

**1.4a** 27,14±4,29 18,57±1,43

**28.2** 12,86±0,71 13,57±0,71

**B-1-2-k** 47,14±12,86 23,57±0,71

**26.1** 21,43±0,71 26,43±9,29

**35.2** 67,86±0,71 65,71±4,29

**B.10.3F** 41,43±0,71 42,86±0,71

**B.4.1.i** 47,86±0,71 41,43±0,71

**B.3.5m** 62,86±0,71 12,86±1,43

**10.4.i** 45,71±2,86 42,86±0,71

**24.4.i**  15±6,43 -

^1^Lines in bold are strains that were decided to proceed to the next stage of the study.

^2^-: Ineffective growth (%0 groth inhibition)

^3^The numbers given in parentheses next to the isolate codes are the gen bank accession numbers.

**Table S2.** Growth Inhibition Ratios of Pseudomonas spp. against Trichoderma strains.

**% Growth Inhibition**

***Pseudomonas* sp*. T. aggressivum f. agressivum T. aggressivum f. europaeum***

**P-1.1-b** - -

**P-1.2-a** - 17,86±2,14

**P-1.2-b (MW476562)** - -

**P-1.2.*-a** 9,29±1,43 -

**P-1.4-a**  - -

**P-1.4.*-b** 32,14±3,57 10±4,04

**P-1.4-b-c** 40±2,02 43,57±0,72

**P-1.4.*-c-a** - -

**P-2.6-a-a** 5,71±5,71 7,14±7,14

**P-2.6.*-b-a-1** 18,57±1,43 5,71±5,71

**P-2.6.*-b-a-2** - 17,86±2,14

**P-2.6.*-b-c** - 23,57±9,28

**P-2.6.*-d** - -

**P-2.7-1**  - 22,14±9,28

**P-2.7-2** 3,57±1,80 3,57±3,57

**P-3.1-b**  - -

**P-3.1-c** - 22,14±0,72

**P-3.1.*-b** 6,43±6,43 -

**P-3.3.*-a** - -

**P-3.3.*-b** 14,29±7,14 -

**P-4.3 (MW476568)** - -

**P-4.4**  10,71±0,72 10,71±0,72

**P-5-1**  5,71±5,71 -

**P-5-2** 4,29±1,43 15±9,09

**P-5.6-b** 14,29±4,29 -

**P-5.6.***  39,29±3,58 19,29±2,14

**P-5.6-d**  - -

**P-5.6.*-b-a** 39,29±3,58 25±2,02

**P-5.6.*-c-1**  33,57±0,71 24,29±1,43

**P-5.6.*-c-2**  - -

**P-5.6.*-d-a** 21,43±0,72 5,71±5,71

**P-5.6.*-d-b** 22,86±0,72 5,71±0,72

**P-6.5-c-1** - -

**P-6.5-c-2** 23,57±2,14 -

**P-6.6-1**  24,29±2,15 14,29±4,28

**P-6.6-2**  - 5,71±5,71

**P-8.5-b-1** 20±7,07 9,29±6,43

**P-7.7.*-1** - -

**P-7.7.*-2** - -

**P-9-b**  14,29±1,43 -

**P-9.7-b-2** - -

**P-10.5.*-a** - -

**P-11.7-a** - 15±1,48

**P-12.4-a-1** - -

**P-12.4-a-2** - -

**P-12.5**  18,57±1,43 2,14±0,71

**P-12.5.*-b** 16,43±16,43 7,14±0,72

**32**  43,57±0,72 43,57±3,57

**39** 46,43±0,72 45,71±0,72

**60** 45±2,14 41,43±0,72

**168**  35,71±1,43 35,71±0,72

**176**  42,14±6,43 46,43±3,57

^1^-: Ineffective growth (%0 groth inhibition)

^2^The numbers given in parentheses next to the isolate codes are the gen bank accession numbers.
